# Energy Management Expert Assistant, a New Concept

**DOI:** 10.3390/s21175915

**Published:** 2021-09-02

**Authors:** Matias Linan-Reyes, Joaquin Garrido-Zafra, Aurora Gil-de-Castro, Antonio Moreno-Munoz

**Affiliations:** Departamento de Ingeniería Electrónica y de Computadores, Escuela Politécnica Superior de Córdoba, Campus de Rabanales, Universidad de Córdoba, Edificio Leonardo Da Vinci, E-14071 Cordoba, Spain; p22gazaj@uco.es (J.G.-Z.); agil@uco.es (A.G.-d.-C.); amoreno@uco.es (A.M.-M.)

**Keywords:** home energy management systems (HEMS), Internet of Things (IoT), artificial intelligence (AI), Voice Assistant, machine learning (ML), big data

## Abstract

In recent years, interest in home energy management systems (HEMS) has grown significantly, as well as the development of Voice Assistants that substantially increase home comfort. This paper presents a novel merging of HEMS with the Assistant paradigm. The combination of both concepts has allowed the creation of a high-performance and easy-to-manage expert system (ES). It has been developed in a framework that includes, on the one hand, the efficient energy management functionality boosted with an Internet of Things (IoT) platform, where artificial intelligence (AI) and big data treatment are blended, and on the other hand, an assistant that interacts both with the user and with the HEMS itself. The creation of this ES has made it possible to optimize consumption levels, improve security, efficiency, comfort, and user experience, as well as home security (presence simulation or security against intruders), automate processes, optimize resources, and provide relevant information to the user facilitating decision making, all based on a multi-objective optimization (MOP) problem model. This paper presents both the scheme and the results obtained, the synergies generated, and the conclusions that can be drawn after 24 months of operation.

## 1. Introduction

The constant advancement of ICT opens up great opportunities to improve systems’ functionality, performance, and efficiency. Technologies such as IoT, Big Data, AI, WIFI 6, or 5G could come together to enhance the capabilities of equally emerging systems, oriented for use in the home, such as HEMS and Voice Assistants.

The residential sector is a key element in the context of both energy savings and people’s well-being. The restructuring of the energy sector through Smart Grids and Microgrids [1], as well as the arrival of the 5G network [2,3,4] and WIFI 6, will allow an exponential development of connected devices and appliances, opening the doors of the network to the IoT both in the industrial sector and in the home [5].

Bringing all these elements together in the home environment can be challenging, but they form the fundamental structure of the Home Energy Management Expert Assistant (HERMES) system. However, it can offer us a revolution that can positively impact climate change, energy efficiency, or quality of life. Each of these elements separately already offers solutions to specific problems. The literature shows considerable evidence of this use and its benefits, as shown below, focusing on the most relevant, emphasizing the latest efforts and advances in applying the methodologies.

### Home Energy Management Systems (HEMS)

HEMS are hardware and software systems that enable advanced control of energy-using systems and devices in the home, continuously analyzing data to provide real-time information on the energy performance of the home [6], creating data streams (both external and internal such as weather, electricity price, or sensors) and making decisions for energy efficiency improvement [7], peak demand management and demand response, so their specifications include the necessary integration (monitoring and control) and communication with all smart home devices, sensors, relays, and appliances regardless of their communication protocols [8]. A proper implementation would allow a reduction of about 35% of the total electricity bill, prioritizing load consumption based on the cost of energy [9]. Other studies, such as the ACEEE study not focused exclusively on energy cost, set this saving at a maximum of 17% [7].

Nevertheless, in practice, these systems have some limitations, mainly including interoperability between devices, lack of training of the users themselves, doubts about security or limitations of commitment to the customer, as well as the lack of studies showing the real possibilities of savings [5,7,10,11], as well as lacking true intelligence and the ability to manage demand peaks and demand response. To develop an efficient HEMS, it is necessary to know the characteristics and requirements of each of the technologies that will allow a complete communication and configuration of all the devices [12,13,14,15]. One solution to interoperability would be implementing a widely consolidated Building Management System (BMS) [16]. However, they are very closed systems, mostly proprietary solutions that can only be upgraded by the system manufacturer with relatively high costs, ranging from $25 to $70 per square meter of housing [17]. On the market, we can find a wide offer of both Open-Source and Proprietary HEMS [8,18]: Open-Source Home Assistant [19] and OpenHAB (based on Eclipse SmartHome™) [20,21] that have a large number of protocols and configurable devices, BEMOSS [22] built-in Python on VOLTTRON [23], ioBroker [24], Open Energy Management (OGEMA) [25,26], and Open remote [27], among others.

**Figure 1:**
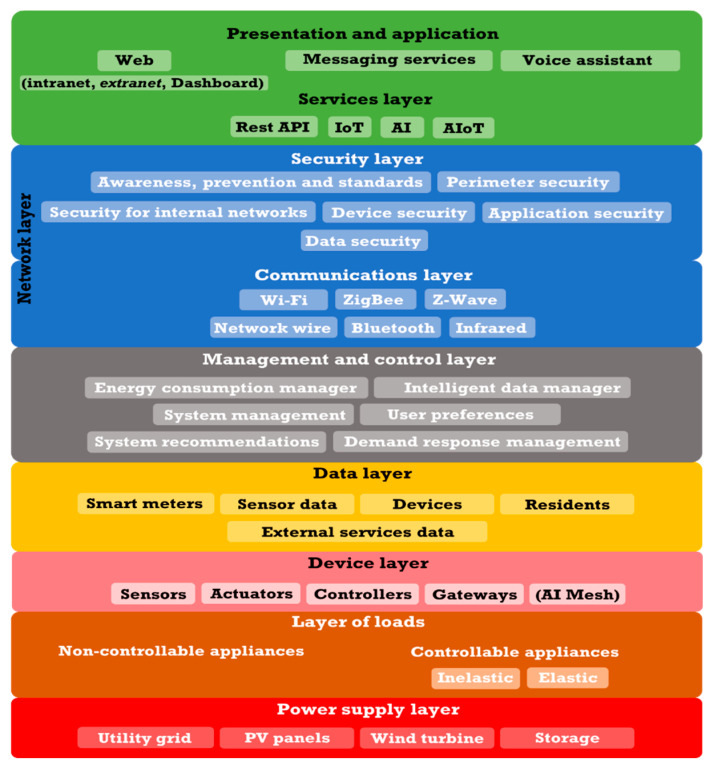
The generic architecture of a HEMS system.

In this paper, we will consider the following architecture of an advanced HEMS system (Figure 1):

We continue in the next section by detailing the main elements that will make up our HERMES system.

## 2. Materials and Methods

In addition to the energy management system described in the introduction, the HERMES system is developed integrating the following elements:

### 2.1. IoT: Smart Devices

Depending on the context, there have been many definitions of IoT [28,29,30,31]. A good approximation to its current definition could be: “System of devices, machines, or everyday objects provided with unique identifiers (UID) with the ability to transfer data through a network without the need for interaction between people or between people and computers”.

In recent years the number of Internet-connected devices has exploded, to such an extent that forecasts have become outdated [32]. There is currently no consensus on their number, offering figures that range from CISCO’s 50 billion [33] to Intel’s 200 billion [34]. In parallel to this growth, their price has been reduced by more than 90%, coining the concept of “democracy of devices” [35].

Not only devices or things but also household appliances are beginning to join the world of elements connected to the network, and its growth will be almost total in the coming years with the arrival of 5G and the IPv6 protocol without address exhaustion problems (2^128^ addresses).

Therefore, IoT is a significant challenge for HEMS as it has to interact with a large number of “smart devices” with a wide variety of protocols, in addition to the large amount of data they will generate. In this regard, some emerging technologies can help address this challenge: big data, cloud computing, and AI, as also postulated in [17,36].

### 2.2. Big Data

Traditionally big data refers to the concept of an amount of data that exceeds the capacity of conventional software to be captured, managed, and processed in a reasonable amount of time. Nowadays, the concept extends to analyzing user behavior, extracting value from stored data, and formulating predictions through the patterns observed. A first approximation to this definition was given in 2012 by [37].

Big data uses the following characteristics described by the three V’s: volume, variety, and velocity, and several other characteristics including veracity, value, and the identification of nonlinear systems (from large data sets) to reveal relationships or to make predictions of outcomes and behaviors [38,39,40].

From the large amount of data generated in a smart home, whether internal (through the IoT network integrated into the home) or external (such as weather or electricity prices), it would be interesting to improve energy efficiency, to analyze these data and extract all the relevant information they can provide to the system, so the use of big data can be an essential tool, providing great value for the optimization of home resources and user comfort. However, the storage, processing, and analysis of this large volume of continuously generated data, while maintaining their security and privacy, is a significant challenge for HEMS.

To this end, HERMES uses various strategies to process volumes of data, store the information periodically and in real-time, and process it to obtain an analysis and projection of the data to trigger specific automated actions without user intervention. It also offers information that is provided to the user through the Expert Assistant to guide decision making or as information on predictions or patterns detected through machine learning (ML).

### 2.3. Cloud Computing

Cloud computing is the resources and services of the computer system accessed through a network without direct active management by the user. Initially, the services were focused on data storage and computing power, but, today, the user’s services and systems cover virtually any need.

Cloud computing offers advantages and disadvantages that must be assessed by the user when implementing or not implementing these systems. From the point of view of HEMS, we can highlight the following advantages:

- Reduction of costs and implementation times;
- Reduction of scalability problems in cases where the system must grow;
- The user can focus on the system’s functionality and not on the technical aspects of the infrastructure;
- Access to services from anywhere;
- System portability and protection against data loss. If the local system suffers damage or failure, the data or services in the cloud remain secure and loss-free;
- Transparent updates for the user, as long as the vendor maintains this commitment and the local system is not affected by version incompatibilities;
- Software installation is avoided or reduced;
- Local system requirements in terms of computational capacity are reduced. By deriving computing services and processes to the cloud, a lighter hardware system is required. This, in turn, leads to a benefit due to reducing local consumption by requiring equipment with lower performance;
- Security is often a critical factor in these services as providers can equip their systems with the latest technologies in the face of the limitations faced by a single user, both in terms of technological capacity and knowledge.

On the contrary, some drawbacks can be very critical to the viability of the system:

- Absolute dependence on the commitment or continuity of the service provider: discontinuity or modification of services may critically affect the HEMS system;
- Fixed fee for the use of the services;
- Lifetime dependence on external suppliers;
- Small systems are more vulnerable than more extensive infrastructures, especially concerning:
  ○ Downtime;
  ○ Technical interruptions from suppliers, which are unavoidable and can occur at critical moments;
  ○ More limited bargaining power, leading to limited customization;
- Dependence on external network access versus a HEMS system based on a local network isolated from the Internet;
- Aspects such as security, privacy, or confidentiality may be exposed or compromised.

Although a priori cloud computing is possibly an inevitable tool if we want to develop a truly competitive HEMS, we must be very aware of some of the limitations and implications that its use may entail, so we must adopt hybrid strategies between functional HEMS through local networks isolated from the Internet and HEMS based on cloud computing. Therefore, we are committed to systems that take full advantage of the functionality of the isolated network and to ensuring that the contracted services, which are based on or use the Internet, do not pose a risk or functional disruption to the system.

In this regard, the design of the HERMES system presents a dual functionality with communicating vessels between the own network (partially isolated) and the contracted cloud computing services. In addition, to protect the system’s security, various levels of protection have been planned according to its exposure to the Internet. HERMES can maintain operational functionality if, for security reasons, it is decided or required to isolate the system from the Internet.

### 2.4. Artificial Intelligence (AI), Expert System (ES), and Machine Learning (ML)

AI is the ability of a man-made system to interpret and analyze data, learn from that data, and use that new knowledge to perform actions or tasks. This definition is an evolution of the one given by Andreas Kaplan and Michael Haenlein [41]. Another agent-based approach defines it as: “Computational intelligence is the study of the design of intelligent agents. An intelligent agent is a system that acts intelligently: What it does is appropriate for its circumstances and its goal, it is flexible to changing environments and changing goals, it learns from experience, and it makes appropriate choices given perceptual limitations and finite computation” [42]. The definition is not trivial and has evolved over the years to encompass very diverse disciplines with applications in virtually all scientific fields [41,42,43,44,45,46], such as expert systems that emulate the behavior or responses that a human expert in an area of knowledge would give.

**Figure 2:**
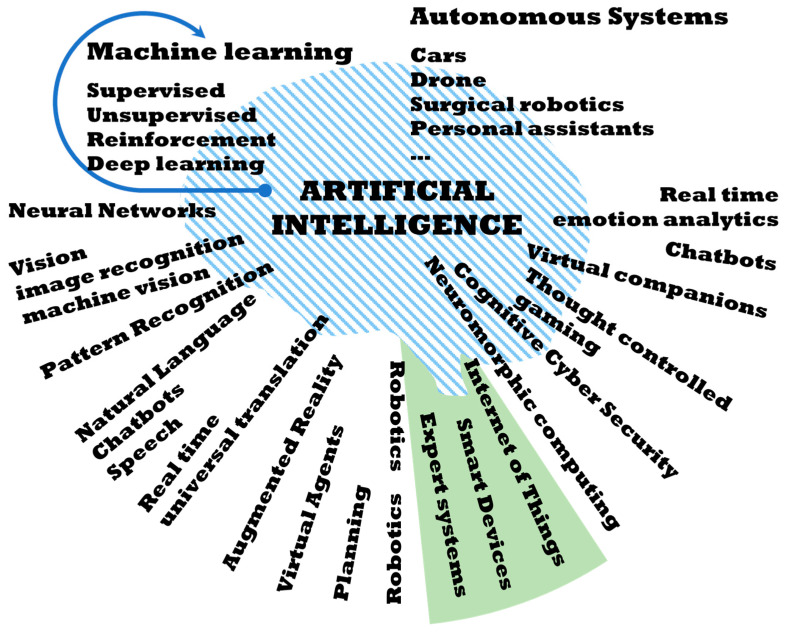
AI Technologies.

There is no doubt that IA is a fundamental tool in the present and future development of HEMS. IA encompasses a multitude of technologies, some of which are shown in Figure 2:

The HERMES system has been developed integrating ML, natural language processing, expert systems, and speech, without ruling out other technologies in the future such as image recognition (vision) for more advanced analysis of presence [47,48,49] with a higher level of personalization of interactions.

### 2.5. Virtual Assistant

A Virtual Assistant [50] or Voice Assistant is a software agent that can interpret human speech and certain commands and respond with synthesized voice, tasks, or services. Other definitions can be found in M. B. Hoy [51]. The development of natural dialogues between humans and machines is one of the goals of AI [50,52]. Voice assistants are here to stay [53], not only because of their benefits for people with specific needs or older adults [54,55,56,57] but also because they have been shown to bring benefits such as social cohesion [58] or improve comfort and allow the user to interact in a very natural way with machines, since speech is the main mode of communication for humans [59]. In our case, the last of the pillars that make up the HERMES system is precisely the assistant but endowed with greater intelligence, as we will indicate below.

**Figure 3:**
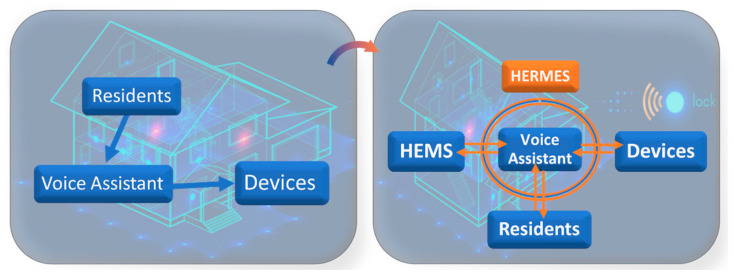
HERMES: Bi-directional dual-channel formed between HEMS-Voice Assistant-Residents. Background: © 123fr.com.

From a HEMS perspective, voice assistants have several notable handicaps. First, their intelligence is limited in terms of energy efficiency, as verbal commands and functionality are focused on activating or deactivating devices. However, our HERMES system integrates a bidirectional communication channel to the virtual assistant (Figure 3) both with the system as a whole and with the residents, connecting the intelligence of the system with the user, becoming an “intelligent assistant” beyond the function and intelligence of these systems, complementing the functionality of the HEMS. This dual bidirectional channel represents a qualitative leap in the functionality and interaction of the HEMS system with the users.

Secondly, another important handicap is the vulnerability presented by these devices; for example, any user can issue verbal commands: “Open the door” or “buy this and send it to such address”) [60,61,62]. In this case, the integration of the voice assistant in the HERMES system is done keeping in mind that this type of vulnerability cannot be fraudulently passed on, the system itself is the one that filters them. In this regard, HERMES detects the presence at the home of all the usual residents and identifies them so that certain commands can only be executed if at least one of them is at home or if the presence simulator has been activated, which can be activated remotely by the residents for a limited time. Other avenues that could be explored to avoid vulnerabilities could be the identification of users by smart cameras or by their voice profile [63,64]. In this way, all commands, or those that we consider critical to the system, can be filtered to prevent a local or network intruder from exploiting them.

### 2.6. Results from Knowledge

The benefit of applying advanced and complex systems must be realized from the knowledge acquired from the collection of data and the application to balanced models, not forced, that allow the creation of precise and effective forms counting at all times on the users. Otherwise, the system will lack practical application, falling into the dynamics of a good theoretical study without a practical route. Therefore, the system has been developed in different phases, data collection being the first of them, from which practical solutions have been channeled. For this reason, the system has been developed in different phases, the first of which is data collection. Based on the data, practical solutions have been proposed, focusing on economics but adapting to the users, which allows long-term habits to be established, quality of life to be maintained, and ensures that the system is applied as it is beneficial.

From this dynamic of results from knowledge, the integration of all these systems in one (HEMS + IoT + Big Data + Cloud Computing + AI + Voice Assistant = HERMES) has led us to an ES with a multitude of possibilities in the field of energy efficiency and well-being of residents. This work shows the development of a new system with the following objective: “developing a comprehensive model for smart home consumption management assisted by an ES (HERMES)”.

The development of this objective was based on the following pillars: results from knowledge, energy savings, usability, user assistance, comfort, privacy, and security. The following lines of analysis and development were proposed:

- Obtaining data to develop the best possible system for the objectives pursued;
- Cloud integration provides the system with scalable computational capacity, access and management of information flows, extension to big data analysis, AI, and ML;
- Usability: “Home” system of interaction with the user that allows triggering complex services from a simple and accessible functionality:
  ○ Actions: Programming household appliances and devices. Presence detection and habit analysis;
  ○ Warnings: Advice and recommendations for savings based on detected habits or pre-established patterns;
  ○ Alerts and maintenance of equipment and appliances;
  ○ Integration with voice technology.
- Measures to ensure user privacy and system security;
- Interoperability of electronic systems that allow the implementation of the integral model;
- System specifications to enhance efficient energy consumption management under IoT architecture;
- Estimated energy savings and user benefits. Differences between HERMES, HEMS systems, and a non-connected home.

## 3. Materials and Methods

It is not easy to make accurate predictions of electricity demand, microgeneration, or appliance usage in domestic environments. Factors such as the type of billing (five main energy billing approaches can be found in the literature [65,66,67]), weather conditions, or assumed habits and routines of users involve in themselves elements of uncertainty that are difficult to predict, so that deviations on forecasts of electricity consumption, microgeneration, or the operational needs of household appliances, can compromise the planning of HEMS. These uncertainties may result in situations where contracted power limits are required to be exceeded with consequent limitations or penalties, or the comfort level of residents may be affected. Therefore, in decision making, the value of past and present data must be prioritized over future data, with dynamic (stochastic) programming approaches [65,68,69].

If we add to this uncertainty the diversity of load types and their different scheduling possibilities, HEMS design strategies can be approached from multiple perspectives [65,68,69,70,71]. Before focusing in more depth on our development, we will review some of the discussed aspects to settle and show the fundamentals of the HERMES system presented in this paper.

### 3.1. Classification of Load Types

There is no consensus on the classification of load types, so we propose a new model that will be useful for our work and is based on several classifications that focus on the characteristics of the loads [65,67], but to which we add the user’s decision capability through the wizard or by programming so that some devices can change category based on the user’s decision.

**Figure 4:**
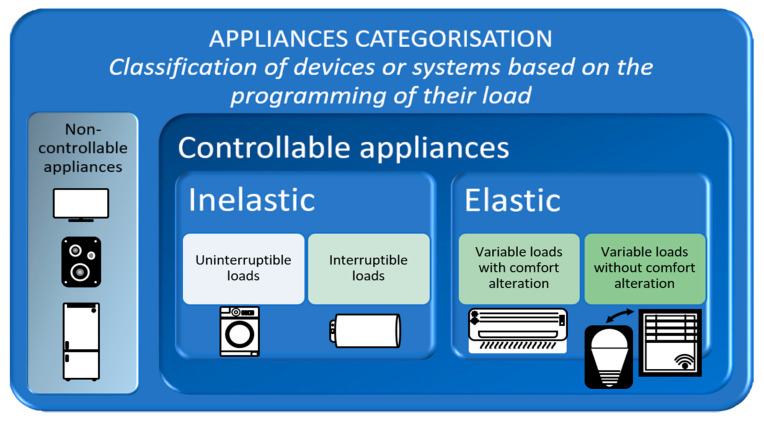
Classification of equipment or systems based on the programming of their load.

Classification of devices or systems according to their load scheduling (Figure 4):

1. Non-controllable loads. Their operation cannot be programmed, changed, or reprogrammed by a HEMS. They usually provide added value, and users control some of them. Televisions, stereos, computers, or appliances such as refrigerators or lighting without control systems fall into this category. Appliance standby would be included in this class;
2. Controllable loads. The HEMS system has some control over them or through the user in a given time horizon. A traditional HEMS system could not control the loads through the user; in this regard, control is one of the contributions of the HERMES system. In turn, within this category, we can divide the loads into elastic or inelastic;
  a. Inelastic. Once initiated or required to operate, it must complete a full cycle;
    i. Uninterruptible loads. Once started, they must run a complete cycle continuously; only the corresponding start time can be programmed. In this category, we can find dishwashers, washing machines, or dryers, among other appliances;
    ii. Interruptible loads. Once started, they can be interrupted but must be reconnected to complete the full cycle. These are usually constant-drain devices. Examples include plug-in hybrid electric vehicles and other rechargeable devices or external batteries, and the electric boiler;
  b. Elastic. Loads with the capacity to be able to adjust power consumption in the middle of an operation;
    i. Variable loads with alteration of comfort. Energy consumption can be adjusted in the middle of an operation but leads to loss of comfort and may require subsequent compensation. These are usually systems whose operation is maintained according to a reference defined by the residents, so their temporary variation by the HEMS may affect comfort. Ventilation, heating, or cooling are examples of this category;
    ii. Variable loads without alteration of comfort. Energy consumption can be adjusted in the middle of an operation without significant loss of comfort or subsequent compensation. For example, dimming of artificial lighting by compensating with daylight.

In the HERMES system, the above examples of appliances could change category (temporarily or permanently) depending on the user’s decision-making. An extreme example could be the refrigerator defined a priori as an uncontrollable load. The user can instruct the HEMS assistant to turn it off for a short period that does not jeopardize food preservation, making it controllable, inelastic, and interruptible. This example can be used to avoid a peak demand as long as there is no other controllable load to bridge the peak demand.

Based on the above structure, Table 1 is a classification of the main household appliances.

This classification is flexible and dynamic since the system can adjust specific parameters according to the characteristics of the residents or according to different scenarios. The system has general and appliance-specific parameters that it can readjust (see Table 2) to adapt to a dynamic classification of appliances. In certain cases, this adjustment is shared by the system and the users, as could be the case for the air conditioning temperature. This behavior thus allows the system to adapt to the characteristics of different user groups and different scenarios (seasons of the year, vacation absences). Users can adjust these parameters within a range and even set the air-conditioning switch-on temperature by voice. The system acts accordingly to maintain comfort but reduce consumption, for example, after a period of operation, raising or lowering the cooling/heating temperature.

### 3.2. The Preamble of the HERMES System

HERMES system scheduling is performed to manage a present and future time horizon based on past and present data. Both a continuous representation of time and a discretization into the minute, hourly, daily, weekly, and monthly intervals are used. For example, once a month, a heating cycle above 60 °C is completed in the electric boiler to eliminate possible Legionella outbreaks. This scheduling pursues the reduction of the consumption of household appliances and the shifting of loads (shifting to optimize expenditure and their optimal time of operation) to reduce electricity billing [72,73,74] and maintain or increase the comfort of residents [73,75]. Regarding billing optimization, the appliance scheduling technique based on mathematical optimization is suitable for small-sized problems such as individual dwellings instead of other less demanding techniques for larger problems, as we will discuss later. By contrast, in terms of comfort, the evaluation of resident comfort is a very complex task from a scheduling point of view due to how personal the perception and subjectivity of comfort can be, as well as the inconveniences of having to schedule appliances outside the preferred time window, maintain a certain order (washing machine before dryer) or accept unwanted elastic load modulations. As a step before implementing the HERMES system (as of 27 October 2019), daily usage profiles were recorded over an extended period (from 16 February 2019 to 26 October 2019) to characterize and minimize potential drawbacks that could affect comfort.

In addition, given that household demand cannot be predicted with complete accuracy, we rely on a consumption profile characterized by minimizing the elements that introduce a certain degree of uncertainty, reinforced by two-way communication with residents to whom, on the one hand, electricity prices are provided a day in advance, as well as other statistics, and on the other, the system analyzes the use of household appliances and recommends their use based on history, coordination, and the use of appliances. This minimizes the problems of stochastic optimization, and although not all the elements that are a source of uncertainty and their derived problems (consumption peaks with penalties or loss of comfort) are avoided, they are reduced, and with experience, the residents themselves and the system learn and converge towards an increasingly optimal situation in terms of billing and comfort. However, deviations of one from the other are allowed, although both are the ultimate goal, so that the system is constantly evolving around the optimal balance at all times, maintaining an MOP [76,77] that is very competitive with other techniques [65] of setting a single objective and the rest as constraints. MOP has allowed us to satisfy both consumer and system objectives [78].

Using this bidirectional technique, which not only brings benefits but has also allowed us to limit uncertainties, it has been possible to implement stochastic dynamic programming with at most two levels of estimation: the target variable plus an additional level with stochastic variables, which greatly increases the accuracy of the predictions as will be seen in the results section. The following references show up to six different strategies for stochastic optimization: stochastic optimization, robust optimization, chance-constrained optimization, stochastic dynamic programming, stochastic fuzzy optimization, and stochastic model, which generates synthetic consumption profiles [65,68,79,80,81,82,83,84,85]. For example, in [79] a stochastic energy consumption scheduling algorithm based on time-varying prices known in advance (similar to the one used in the HERMES system) is described as achieving a 24% to 41% reduction in simulations in billing costs. However, in HERMES, we have opted for a mixed model (with some elements with deterministic programming and others with stochastic programming), which has allowed us to obtain very similar reductions but with real data, not simulated, of up to 42% in absolute values (see Table 8) and 24% with counterbalanced data (see Table 6). Other techniques achieve reductions from 8% to 35% of the electricity bill [9], the optimization-based residential energy management (OREM) technique being the most efficient [86] based on dividing the days into time slots, very similar to the time of use (ToU) scheme and the one proposed in this article, scheduling the operating time of the appliances in the minimum tariff time slot, but in our case minimizing the delays of the OREM technique by shifting the loads in a very efficient way combining the strategy with other techniques.

The various techniques employed in HEMS scheduling to find the optimal operating time of household appliances can be grouped into five categories [65,87]: mathematical optimization; heuristic and metaheuristic methods; model-based predictive control; ML; and game theory approaches. Each of these techniques has strengths for certain types of loads versus weaknesses for all other loads, and in almost all cases, the benefits provided drop drastically when uncertainties manifest themselves in a practical or worst-case form. The main source of uncertainty comes from the residents themselves, who are often influenced by external factors that are difficult to predict (changes in routines, illness, cancellation of a meeting) or varying perceptions and subjectivity. Based on this, HERMES decided to use a mixed model of techniques that would allow the residents to make their own decisions or let the system decide independently under MOP, using techniques such as mathematical optimization, heuristics, or ML.

The subsection “Equations” shows mathematical elements used and developed under a tree structure for decision making and resident assistance. Further on, the mutual learning process between the system and the residents will become evident by adapting the system to the residents’ habits and the residents’ system, which makes it possible to achieve the percentages of reductions indicated above. This feedback has allowed very significant improvements in the first year, which were further improved in the second year and again in the third year. This continuous improvement highlights the bidirectional interaction of the system with the residents, which would be difficult to achieve by applying a single technique and without the expert assistant to interact with and guide the residents.

As indicated, to highlight the potential of the wizard, HEMS has been developed on a mixed model of techniques [87] to improve energy use through load scheduling, in which uncertainties have been minimized so that these models must be able to admit the interaction of several agents that would become the elements of uncertainty as well as load scheduling. The objective of all HEMS is to optimize consumption, so they require scheduling over a future time horizon, for which household demands and electricity generation cannot be accurately predicted, requiring adequate consumption profiles, representative, and incorporating a certain degree of uncertainty management. For all these reasons, their efforts are focused precisely on optimizing consumption profile predictions. In our case, to highlight the assistant’s potential, we have reduced the uncertainties to a scenario in which the development of HEMS is already considered sufficiently mature, with the assistant being a differentiating element and allowing us to show its potential. In this regard, to optimize consumption profile predictions, we will adopt a dark box model (modeling and forecasting frameworks based on data analysis schemes) as opposed to white-box models (classical and transparent modeling tools based on solving physical equations) and gray box models as a combination of white box and dark box [87,88]. We have limited the uncertainties to the demand area without incorporating electricity microgeneration and setting variable but known day-ahead prices. To obtain the prediction of household consumption, the element used for data analysis was based on ML techniques. Other data analysis techniques could have been applied (see [87]). A very accurate and robust model has been obtained using stochastic data of only two levels: The target or output variable plus an additional level on certain input variables of the ML itself. Since the HERMES system combines different techniques, e.g., deterministic programming for MOP or stochastic programming for consumption estimation, we have tried to simplify it, while trying not to harm the pursued objectives, as we will see in the next section.

### 3.3. Deployment of the HERMES System and Involved Instruments

The basis for developing the HERMES system to optimize savings and comfort is collecting past and present data and forecasting certain elements to create a robust and elastic energy use model. The only essential future data are the hourly kW price (€/kWh) 24 h in advance; thus, the chosen tariffs allow us to predict their value quickly; however, if they were not known, they could be obtained from prediction models with very accurate approximations. The weather forecast and the presence of the residents in the home are also necessary but not essential future data.

**Figure 5:**
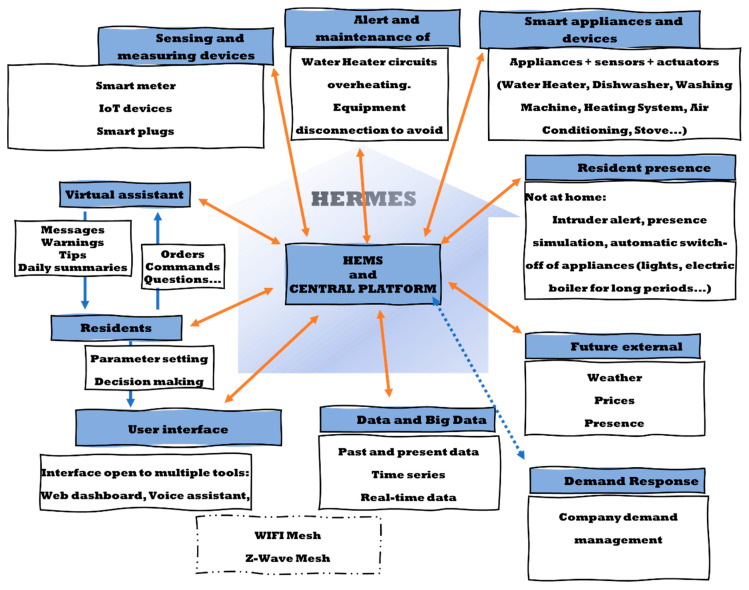
The general framework of HERMES system elements and services.

Given the complexity of our system, and the need to obtain data, its implementation has been gradual, following “natural growth” towards the proposed objectives. Figure 5 shows the principal elements and services of the HERMES system.

Each of the elements shown in Figure 5 has been developed considering analysis, characterization, development of operating models, improvements achieved, deployment of the models and implementation, review of results, and return to the previous phase as necessary.

### 3.4. Programming and Multi-Objective Optimization (MOP) of the HERMES System

**Figure 6:**
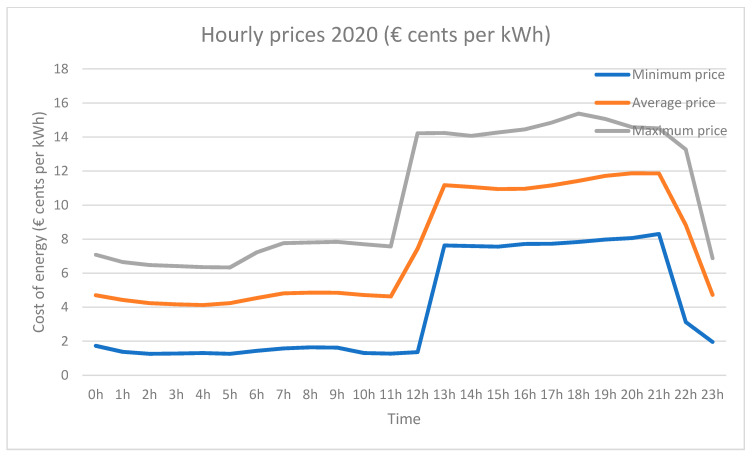
Average hourly prices with the range of fluctuations during 2020.

**Figure 7:**
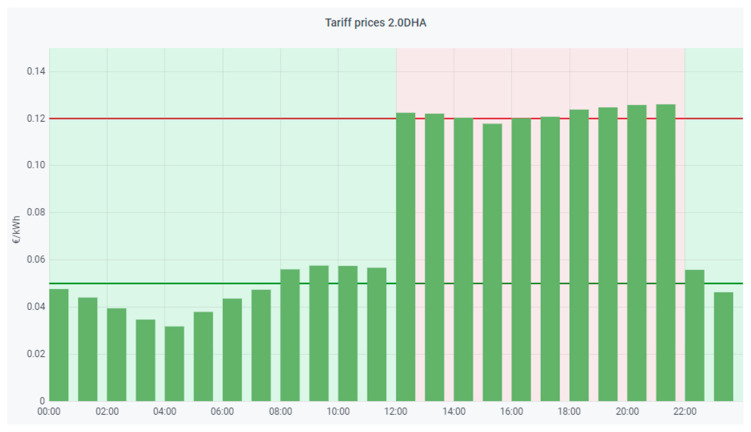
Example graph sent daily to the user with the next day’s prices (example of 11 December 2020).

As discussed above, the HERMES system is based on MOP scheduling in order to (1) reduce electricity bills by reducing appliance consumption and shifting loads and (2) maintain or increase residents’ comfort. The system sends the residents the next day’s hourly rate by instant messaging; they can also consult it at any time through the HERMES user panel or consult it through the wizard. In Figure 6 and Figure 7 we can see different data of the 2.0DHA electricity tariff. Although there are two well-defined time slots, there are significant daily variations in prices for each hour, so the system uses the daily prices in its programming, using any tariff as long as the prices are known or estimated with daily anticipation. For each day, the system selects the optimal time zone.

#### 3.4.1. Equations

HERMES optimal scheduling is modelled as an MOP problem [89]). In this model, the first objective ($f_{1}$) is related to the minimization of the monthly bill, so the scheduling can shift loads either on time scales of minutes, hours, or even between days, with a monthly horizon for the optimization, instead of a daily horizon as most HEMS schedules usually present. Therefore, the shifting of loads is allowed even between days, as long as it does not impair the comfort of the residents, so that the first objective can be formulated as:

(1) $$f_{1}=\overset{PT}{\underset{d=1}{\sum }}\overset{HD}{\underset{h=1}{\sum }}\left[p_{d,h}^{Tariff}E_{d,h}\right]$$

where:

- $E_{d,h}$ = Energy consumed by the household in kWh during the hour of the day h of the day d of the tariff period PT;
- $p_{d,h}^{Tariff}$ = Price in €/kWh of the hourly cost of energy term in each hour of the day h of the day d of the tariff period PT for the contracted tariff (Tariff);

Equation (1) excludes fixed costs and taxes not associated with consumption. The choice of the tariff is important because it determines both the variable costs and part of the fixed costs, so, initially, a study was made to determine which tariff was the most suitable for the habits of the residents and the potential of the HERMES system. This choice led to a first saving in the monthly bill without affecting comfort, as reflected in the results (see the first part of Table 6).

To minimize the value of the function $f_{1}$ several resources and constraints must be considered:

(2) $$P_{a}=P_{a}^{UNC}+P_{a}^{Con,Ine}+P_{a}^{Con,Ela}\leq \{\begin{array}{c} P_{aMax}^{Tariff}\\ P_{aMax,\;penalty}^{Tariff} \end{array}$$

Equation (2) establishes that the active power at any instant of time $P_{a\;}$ expressed in kW cannot exceed the maximum contracted power $P_{aMax}^{Tariff}$ nor the one higher than this one of penalty $P_{aMax,\;penalty}^{Tariff}$ (in the case under study, this limit is set at 105% [90] of the maximum contracted power $P_{aMax}^{Tariff}$). The $P_{a}$ is the sum of all household loads, consisting of uncontrollable loads $P_{a}^{UNC}$ and controllable inelastic $P_{a}^{Con,Ine}$ and elastic $P_{a}^{Con,Ela}$ loads. This constraint affects the HEMS scheduling, which is oriented to avoid reaching the maximum allowed and the penalty level. However, due to the freedom of the residents, in case of reaching the first level (5.5 kW), the assistant warns the residents, and in case of exceeding the penalty level (5.775 kW) the system can act by disconnecting elastic loads, and the warning of the assistant to the residents is of greater emphasis.

One relevant aspect is the ability of the system to schedule shiftable loads within the entire known price period $PT_{Known}$ guaranteeing the comfort of the residents and, extending the scheduling horizon beyond the 24 h with which HEMS normally work:

(3) $$PT_{Known}\geq \;24\;\mathrm{hours}$$

This condition allows the system to increase consumption on days whose prices are lower than adjacent days, i.e., the system reschedules the loads when it obtains the prices for each hour of the following day, also taking into account the prices for the rest of the current day. After obtaining the prices, the system performs sorting by prioritizing the cheapest hours:

(4) $$Array\left(p_{d,d\;+\;1,h}^{Tariff}\right):\;\left[\begin{array}{ccc} Min\left(p_{d,d\;+\;1,h}^{Tariff}\right) & \ldots & Max\left(p_{d,d+1,h}^{Tariff}\right) \end{array}\right]=\left[\begin{array}{ccc} p_{1} & \cdots & p_{m} \end{array}\right]$$

where $p_{1}\leq \cdots \leq p_{m}$; and assigning specific controllable loads (such as the electric boiler $P_{a}^{E.Boiler}$, dishwasher $\;P_{a}^{Dishwasher}$ or batteries $P_{a}^{Baterías}$) to the cheapest hours according to the energy required by each load (for example $E_{E.Boiler}>E_{d,h}>E_{Batteries}$) while maintaining the constraint (2) and comfort:

(5) $$p_{1}\overset{d,h}{\to }\alpha_{11}P_{a}^{E.Boiler}+\alpha_{12}P_{a}^{Dishwasher}+\;\ldots \;+\alpha_{1n}P_{a}^{Batteries}\leq P_{aMax}^{Tariff}\;p_{2}\overset{d,h}{\to }\alpha_{21}P_{a}^{E.Boiler}+\alpha_{22}P_{a}^{Dishwasher}+\;\ldots \;+\alpha_{2n}P_{a}^{Batteries}\leq P_{aMax}^{Tariff}\;p_{m}\overset{d,h}{\to }\alpha_{m1}P_{a}^{E.Boiler}+\alpha_{m2}P_{a}^{Dishwasher}+\;\ldots \;+\alpha_{mn}P_{a}^{Batteries}\leq P_{aMax}^{Tariff}$$

where $\alpha_{ij}$ form a matrix m×n of binary coefficients associated with each controllable load as a function of the energy required by each load ($\alpha_{11}$ is associated with a load whose energy is greater than the equivalent for $\alpha_{12}$ and so on until the $\;\alpha_{1n}$; the next row corresponding to $p_{2}$ represents loads whose work duration extends beyond the hour  h  associated with the minimum price $p_{1}$. The constraint is given by $P_{aMax}^{Tariff}$ forcing the HEMS scheduling to set to zero those loads whose energy is lower, i.e., to those coefficients $\alpha_{ij}$ of higher columns, so that if the power $P_{aMax}^{Tariff}$ is exceeded, they would not be activated until the next cheapest hour or once the appliances with higher loads have finished their operation (or the sum of the loads already allows incorporating a new lower load). An example matrix for three controllable appliances might look like the following:

(6) $$\alpha =\left[\begin{array}{c} \begin{array}{ccc} 1 & 1 & 0 \end{array}\\ \begin{array}{ccc} 1 & 0 & 1 \end{array}\\ \begin{array}{ccc} 0 & 0 & 1 \end{array}\\ \begin{array}{ccc} 0 & 0 & 0 \end{array}\\ \begin{array}{ccc} \vdots & \vdots & \vdots \end{array}\\ \begin{array}{ccc} 0 & 0 & 0 \end{array} \end{array}\right]$$

The operation of the loads is not limited to whole hours, and they can be longer or shorter periods, so the coefficient $\;\alpha_{21}$ set to 1 in this example does not imply that the associated appliance is the second full hour working; it only indicates that it requires more than one hour to complete its cycle. In some cases, the order of the hours is not relevant, whereas in others it is, so the system also takes into account this limitation for each appliance. The matrix also shows that for the first cheapest hour, the system can only activate two controllable appliances to ensure that condition (2) is not violated or that the third appliance requires two consecutive hours to run its program, and that any other sum of consecutive hours would offer a higher sum price. $p_{x}+p_{y}>p_{2}+p_{3}$. Similarly, if during that period residents activate any other loads (uncontrollable, controllable inelastic, or elastic) that compromise condition (2), the system will warn the residents and ultimately displace the elastic loads that can be displaced at that time. In the following results section, Figures 16–18 show how loads of the appliances are concentrated in the least cost hours. Three zones are distinguished corresponding to (1) the zone where the system works without interference from residents, usually night hours; (2) another optimal zone where both the system and the residents activate loads; (3) and a third one associated with residents’ comfort where the system tries not to schedule loads because they correspond to the highest prices and informs the residents through the wizard.

One last remarkable resource to achieve condition (1) has been the development of a cumulative hourly consumption forecast for the next day $\;f_{1,d,h}^{Forecast}$, understood as the expected consumption based on past consumption under similar conditions. It is based on the use of ML linear regression, offering a forecast based on historical consumption data of residents over a long period comprising a total of 9946 h (over 20,000 h for a second version). It uses data such as weather (both historical and forecast data), as well as the percentage of presence of residents in the house, the day of the week and month, and the price of the kWh. The project is developed in the subsection Consumption estimation (Machine Learning), more information is provided in the Data Availability Statement. Based on this forecast, the following can be established:

(7) $$\begin{array}{c} \left(f_{1,d,h}\leq 0.75f_{1,d,h}^{Forecast}\right);\;f_{1,d,h}\leq 0.95f_{1,d,h}^{Forecast}\\ 0.95\;f_{1,d,h}^{Previsión}\leq f_{1,d,h}\leq 1.05\;f_{1,d,h}^{Previsión}\\ f_{1,d,h}\geq 1.05\;f_{1,d,h}^{Forecast};\;(f_{1,d,h}\geq 1.25\;f_{1,d,h}^{Forecast}) \end{array}$$

For each hour of the day, three levels are established that compare the actual accumulated consumption of the day $f_{1,d,h}$ and the accumulated forecast for that same hour $\;f_{1,d,h}^{Forecast}\;$ so that the user can consult through the wizard if their consumption is lower, higher, or close to the forecast. Lower and upper limits are also established in which the system informs the residents through the Wizard without waiting for the consultation; this would be in cases where the deviation is significant, set by default at a deviation of 25% of the expected amount. The residents can modify the margins established in Equation (7). In this way, a reinforcement message is established when consumption is lower than expected and a “warning” in cases where consumption is higher than expected.

The information provided by the ML could also be beneficial in cases where alternative or complementary energy sources or systems to the public power grid are used. We are referring to microgrids in which energy management would be based on a different dynamic and in which generation and consumption forecasting through the ML would become much more important for the objective stated in (1).

#### 3.4.2. Strategy for Comfort and $f_{1}$ Optimization

If the first objective is related to the minimization of the monthly bill $\;f_{1}$, the second objective of the MOP programming of the HERMES system is associated with the residents’ comfort, trying to maintain a balance between both objectives because, on many occasions, they are opposed to each other. Since the HEMS does not have direct access to the uncontrollable loads, both the optimization of function $f_{1}$ and comfort is usually focused on the controllable loads. However, in our case, thanks to the work of the Assistant, residents are more aware of the costs associated with the loads, so there is bi-directional feedback, and the system gains some influence over the uncontrollable loads, allowing optimization of both objectives, $f_{1}$ and comfort more efficiently.

Comfort has a significant amount of resident subjectivity, and its programming can compromise the hardware resources of the system, so to avoid or alleviate these drawbacks, we chose to change the comfort penalty (discomfort) function typically used in HEMS [65,73,91,92] to a parameter approach adjustable by both residents and the system so that residents could vary these parameters to fit their conception of comfort and the system would balance them to optimize the $f_{1}\;$ function within ranges that do not compromise comfort. However, this concept would only be practical if the parameters were associated with each appliance; it would not make sense if they were global, as we would return to the concept of a global comfort penalty function. At the same time, it would not be necessary to define individual comfort functions for each appliance because the residents are part of their programming through the parameter settings, so the programming must be very well calibrated, which requires more extended testing periods in the implementation of the system and a certain flexibility. This strategy avoids the two problems associated with comfort: subjectivity, as users can adjust the parameters within a range, and programming is simplified as it is customized for each appliance; however, it requires a longer testing period.

**Figure 8:**
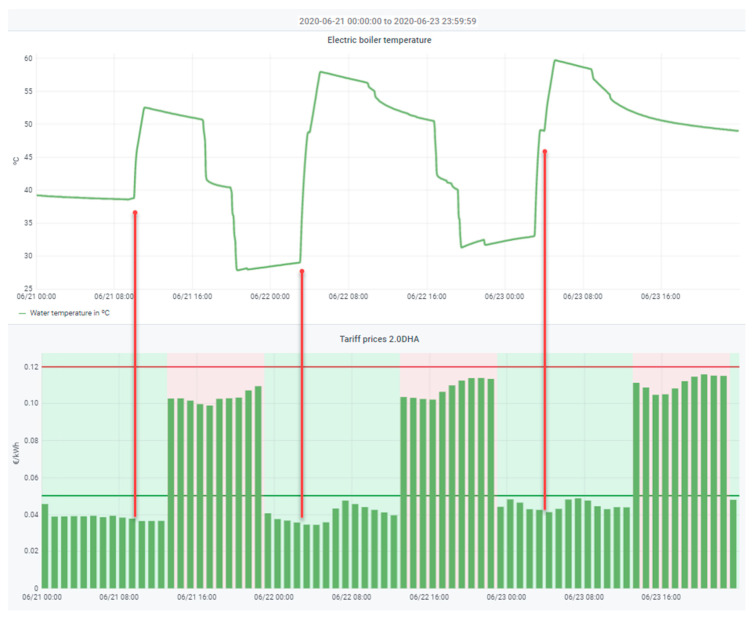
Heating of the water in the electric boiler during the most economical hours. The heating cycle of the boiler is displayed, and the heating hours are marked. Data from 21 June 2020 00:00:00 to 23 June 2020 23:59:59.

The following two examples (responsible for a large part of the electricity bill [9]) show the potential of this approach to comfort: The electric boiler and the air conditioning system:

- The two main problems of the electric water heater are that it runs out of hot water or that it consumes at very high or non-optimal cost hours. In a traditional HEMS, this situation should penalize the overall comfort function, although it might not anticipate the problem or optimize consumption to the maximum. In our case, three groups of parameters have been created to optimize consumption and comfort, solving both problems. The first group selects the time slots in which the thermos flask is allowed to be turned on. The second group sets the temperature targets for each activation band. Finally, the third group adjusts the water heating curve. This third group is continuously adjusted thanks to the temperature sensor inside the tank and determines exactly how long it takes for the boiler to heat the water to the desired values. In this way, if very low temperatures are reached after use, the system responds by increasing the heating time and raising the maximum temperature of each range. The system adjusts these parameters automatically, ensuring the hot water supply and shifting the load to the optimal time slots (see Figure 8). However, residents can readjust the parameters to suit their comfort (maximum heating temperature and the number of heating hours). This set of parameters covers the complete characterization of the water heater, making it possible to cater for particular scenarios such as, for example, completely switching off the electric water heater during prolonged absences by disabling all operating slots, or from time to time run a heating cycle to 60–65 °C to eliminate possible Legionella outbreaks.

- For air conditioning, the HERMES system controls several parameters and employs the following strategy to optimize consumption and maintain comfort: after a certain time after switching on the climate, the system automatically lowers or raises the temperature to reduce consumption while maintaining comfort. The parameters used in this strategy are again three: initial temperature when the heating or cooling is turned on, the time in minutes until the system automatically applies the second temperature regulation (to reduce consumption and which could depend secondarily on other parameters such as outdoor temperature, indoor temperature or whether or not the residents come from outside), and finally, the third parameter would be the difference in degrees of the new temperature. The adjustment of the parameters is again dynamic depending on what the system requires and the residents’ preferences.

In the programming of this strategy for the optimization of the comfort and $f_{1}$ the following parameters associated with loads of each appliance have been used (Table 2):

In the previous paragraphs, the strategies and scheduling of the two main objectives of the HERMES system have been detailed. However, in this multi-objective structure, others could have been added, such as the reduction of $CO_{2}$ emissions in tune with the reduction of the bill, demand response, and others. However, in terms of $CO_{2}$ emissions, any system that minimizes consumption already contributes to reducing emissions, and if it also concentrates consumption in off-peak hours where renewable and less polluting energies tend to prevail, the reduction is even more significant. HERMES provides both benefits.

Therefore, this section can be concluded by indicating that the residents set the comfort levels they desire, and the system optimizes the objectives of minimizing the monthly bill and maximizing comfort, keeping the balance between the two.

## 4. Results

This section shows, evaluates, and interprets the results of the HERMES system. It is developed under the proposed MO model integrating the Assistant.

### 4.1. HERMES System Deployment and Infrastructure

The system has been deployed in a single-family house with four residents with an average annual pre-installation consumption of 6346 kWh and powered exclusively by the electricity grid. The deployment of the HERMES system was carried out in several phases taking into account the characteristics of the house, which has a kitchen, living room, three bedrooms, attic, and three bathrooms.

**Figure 9:**
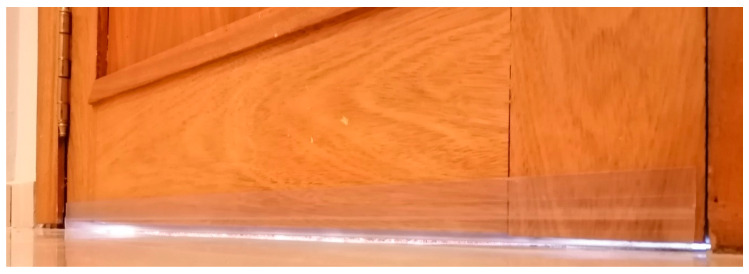
Weatherstripping to improve the thermal insulation of the house.

In the first phase, the passive elements of the house that could affect comfort and thermal insulation were analyzed, something basic but usually a factor that is not taken into account in most installations with HEMS [5]. Some thermal leaks were detected that could be easily solved, such as installing a weather strip (see Figure 9) on the access door to the house, improving the thermal insulation from the outside.

Several groups of sensors, actuators, and various smart home hubs were deployed to achieve efficient control of consumption and comfort in the home. While one group of sensors collected environmental data such as temperature, humidity, or lighting, the second group of sensors collected data on the presence or door opening, and a third group collected data on loads such as power or energy. As for actuators, there were smart switches and sockets for load control. In addition, some appliances already incorporated IoT management. Finally, the various Smart home hubs allowed communication with all sensors and actuators covering various protocols: WIFI, Z-Wave, Zigbee, BLE, and Infrared.

**Figure 10:**
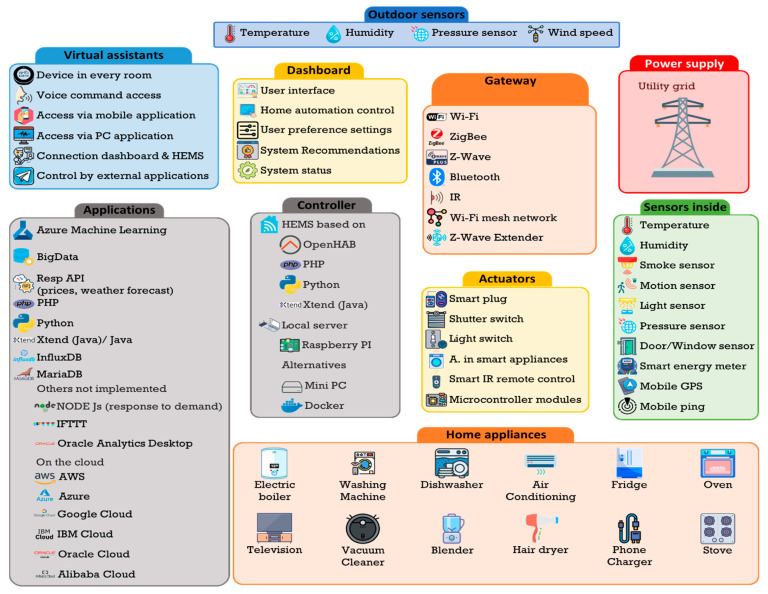
HERMES system infrastructure.

Following the architectural model given in Figure 1, Figure 3, and Figure 5, a comprehensive infrastructure of devices and systems was deployed for the physical implementation of the HERMES system, as shown in Table 3 and Figure 10:

In addition to the sensors and actuators indicated in the table above, the system has sensors for presence, temperature, humidity, twilight, outdoor weather station, door opening in certain rooms and windows, and general consumption meters (energy and power) in the house, as well as consumption meters in certain appliances and meters in two additional areas of the house (lighting + plugs and kitchen).

The deployed infrastructure enables interoperability between devices, event synchronization, real-time (and historical) data logging, analysis and visualization, and present and long-horizon decision making by both the system and the residents, maintaining or improving comfort and cost reduction.

### 4.2. Voice Assistant and Control Panel

**Figure 11:**
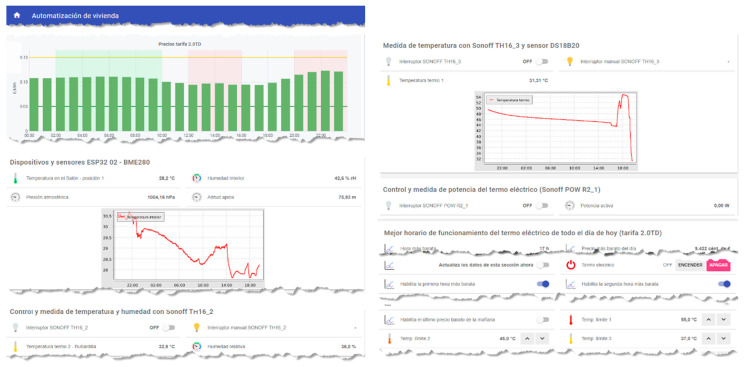
Control Panel excerpt (OpenHAB).

**Figure 12:**
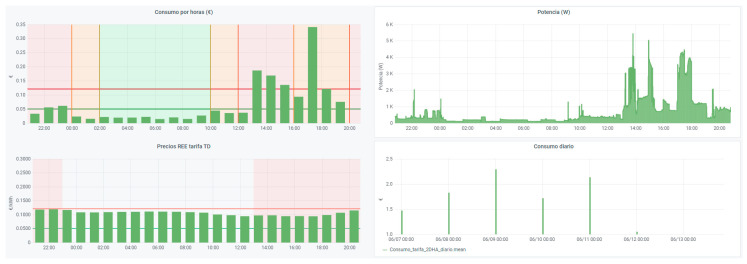
Data dashboard extract (Grafana + InfluxDB + MariaDB).

**Figure 13:**
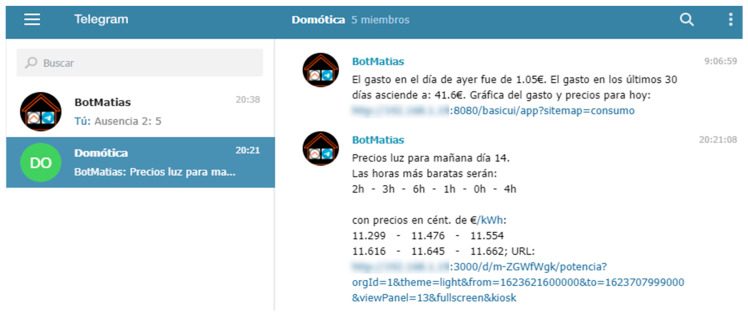
Example of text messages and images sent by the system to residents (Telegram).

The Voice Assistant provides relevant information to residents to safeguard the balance between both objectives ($f_{1}$ and comfort) and accepts voice commands to inform or act on specific subsystems. It is the central core of communication with the residents, although they also have a control panel that offers both information (current and historical data) and the possibility of configuring most of the system parameters. The main interactions of the Wizard (Table 4), an extract of the Control Panel with options for setting some parameters (Figure 11**),** and several data access interfaces (Figure 11, Figure 12 and Figure 13) are detailed below.

### 4.3. Phases of Incorporation of HERMES System Functionalities and Change in Residents’ Habits

Finally, we present a series of data to conclude with the achievements in terms of consumption reduction (cost evolution graphs, load shifting, prices, consumptions, invoices) and comfort improvement (process automation, commands, automated actions).

As we will see later in the subsection “Net load shifting”, residents have a wide margin of improvement for consumption reduction based on shifting controllable (and some uncontrollable) loads to hours with lower prices. The system will try to approach the state of minimum consumption while maintaining comfort. Residents are provided with more information to make decisions they might not have considered before, allowing them to achieve an optimal balance between comfort and electricity bills by adjusting the parameters to their preferences at any time. The information provided by the system through the information panels, or the Voice Assistant keeps users constantly informed of the influence of their consumption habits on their electricity bills.

**Figure 14:**
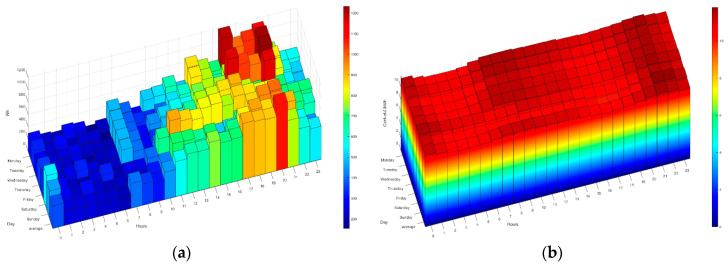
P0 and P1. (**a**) Average consumption (Wh) and (**b**) average prices (€ cents per kWh) during P0 and P1 (tariff 2.0A).

**Figure 15:**
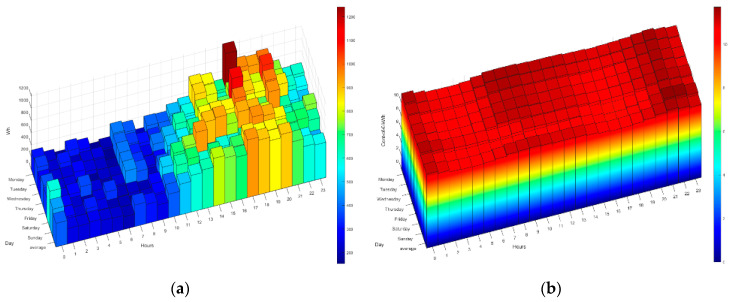
P1. (**a**) Average consumption (Wh) and (**b**) average prices (€ cents per kWh) during the first period (tariff 2.0A).

**Figure 16:**
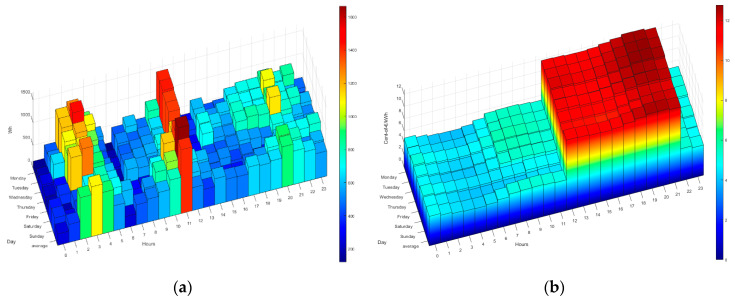
P2. (**a**) Average consumption (Wh) and (**b**) average prices (€ cents per kWh) during the second period (tariff 2.0DHA).

**Figure 17:**
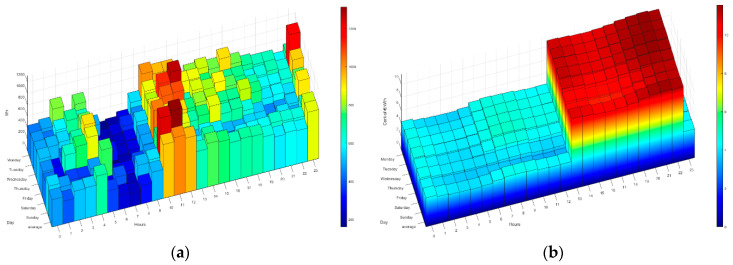
P3. (**a**) Average consumption (Wh) and (**b**) average prices (€ cents per kWh) during the third period from 29 March 2020 to 24 October 2020 (tariff 2.0DHA).

**Figure 18:**
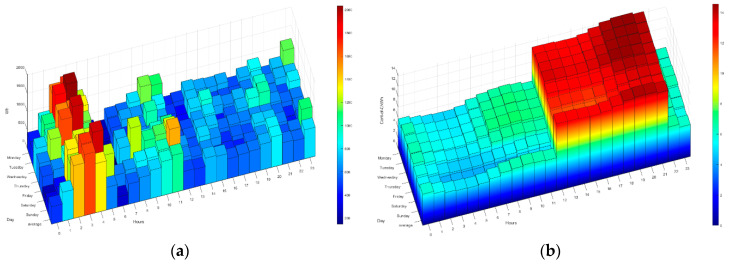
P3. (**a**) Average consumption (Wh) and (**b**) average prices (€ cents per kWh) during the third period from 25 October 2020 to 06 February 2021 (tariff 2.0DHA).

The following figures (Figure 14, Figure 15, Figure 16, Figure 17 and Figure 18) show how the daily distribution of loads has changed in line with prices and the impact these changes have had on bills. Both the system and the residents have been adapting to each other to achieve the above-mentioned optimal balance. The data have been divided into four periods (see Table 5): (0) P0 or previous. (1) P1 or first period where the system was still being implemented, and the optimal tariff was determined according to the residents’ habits and HERMES’ potential. In this period, the system did not yet allow load shifting, but it did offer information on their consumption. It was determined that it was necessary to move from the 2.0A tariff without time discrimination to the 2.0DHA tariff, distinguishing two price bands. (2) P2 or the second period starts with the new tariff, consumption management and allows the displacement of some loads. (3) P3 or third period where the system is implemented with the total operational capacity to displace all dispatchable loads and is ready to readjust the cooling/heating temperature to optimize consumption and comfort managed by the Wizard.

During P1 (first period), the HERMES system infrastructure was developed and started to work effectively from P2 (second period), with full development in P3 (third period). During P1, the residents already have information on their consumption, but the system cannot shift loads. The maximum consumption coincides with the most expensive hours. The pattern of P2 and P3 is very different from that of P1 (see Figure 14, Figure 15, Figure 16, Figure 17 and Figure 18), mainly due to the shifting of loads to the cheapest price hours, optimizing the monthly electricity bills as shown below (see Figure 24). Consumption has shifted from being centered from 17 h to 20 h, coinciding with the most expensive hours, to being divided into two and three bands of specially reduced prices, centered from 02 h to 04 h, from 10 h to 12 h, and 23 h, coinciding with the average of the lowest prices. Above all, this adjustment stands out in the third period, where the load shifting is optimized to reduce the bill while maintaining comfort, being very significant to see how the consumption needs are reduced in the most expensive hours (from 19 h to 21 h) and concentrated in the cheapest ones while maintaining a certain balance due to the maintenance of the residents’ comfort.

### 4.4. Net Load Displacement

It would be necessary to compare the real load distribution with respect to a scenario with no load shifting and no change inhabits to quantify the savings provided by the Hermes system. From the recorded data, two scenarios can be distinguished, one formed by periods P0 and P1 in which there were no load shifts or changes in habits, and another scenario formed by periods P2 and P3 in which HERMES has carried out load shifts, and there is some adaptation of the residents’ habits to the time slots with lower prices.

**Figure 19:**
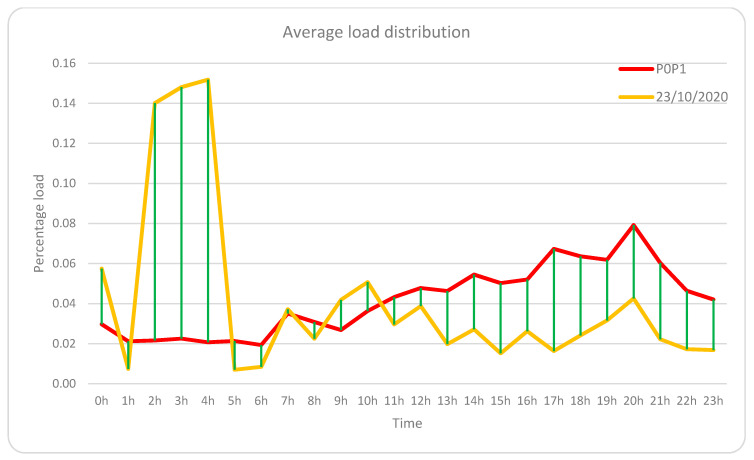
Average load distribution for each hour of the day and the scenario without load shifting or habit adaptation (P0P1 series). The load distribution for day 23 October 2020 has been added as an example to obtain the net load shifting.

From the first scenario (no-load shifting and no change in habits), an “average load distribution” has been obtained for each hour of the day so that the load shifting for any given day can be calculated by obtaining the difference of loads to the average distribution. A distinction is made between shifts that produce savings (above average loads at economic hours or below average at expensive hours) and those that do not produce savings (below average loads at economic hours or above average at expensive hours). The difference between the displacements that produce savings minus those that do not produce savings gives us the measure of the net displacement of loads, this being positive when savings are produced and negative when cost overruns are produced, and the greater the displacement, the greater the savings, balanced by the price per kWh and total consumption, so although it offers a measure of displacement, it does not offer a direct measure of the savings that will be calculated as will be explained later. Figure 19 shows the “average load distribution” for the scenario without load shifting, and the load distribution for the day 23 October 2020 has been added as an example to obtain the net displacement for that day:

The calculation of the “net load shifting” (shifts that produce savings: Add; shifts that produce cost overruns: Subtract) for that day, following the procedure indicated in the previous paragraph, the net balance is positive and has a value of 10.69 kWh:

- Savings-producing displacements: Above-average loads at economical hours or below-average loads at expensive hours;
- Commuting that does not produce savings: Below-average loads at inexpensive hours or above-average loads at expensive hours;
- Economic hours for the day 23 October 2020: 0 h–12 h and 23 h;
- Expensive hours for day 23 October 2020: 13 h–22 h.

**Figure 20:**
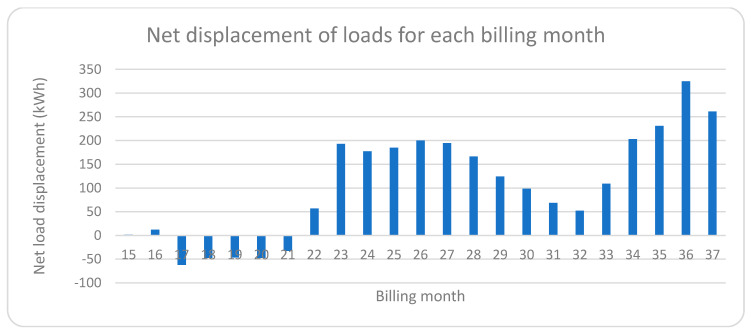
Net load shifting (kWh) per billing month. A positive net balance is obtained from billing 22 due to the performance of the HERMES system.

Suppose we extend this calculation to all the days of the different billing periods (periods indicated in the first column of Table 6). In that case, we obtain the following graph with the net load shifts per billing month, obtaining an average daily net shift of 5.61 kWh for the billing range 23–37, which is equivalent to 35.8% of the average daily consumption established at 15.68 kWh (481.88 kWh for each billing month). In Figure 20, two zones can be distinguished, one with negative shifts where there were no savings and covers from invoice 15 to 21, and the other from 23 to 37 where all net shifts are positive, which indicates the correct operation of the HERMES system. Even invoice 22 already has a positive shift, although it was not enough to obtain significant savings; that month was the one in which HERMES started operating. It is also shown how during the summer months of July and August (invoices 31 and 32), the system is less efficient since the most intense use of refrigeration coincides with the most expensive hours and represents a significant part of the total consumption.

### 4.5. Calculation of Balanced Savings Obtained by HERMES

Once the net load shifting has been obtained, the savings calculation will partly follow the data obtained previously, but taking into account the total consumption of each day and the prices for each hour of that day. We started from the scenario with no load shifting or change of habits, in which electricity tariffs did not influence residents’ habits since the behavior pattern was based on comfort. Based on this pattern, the actual daily consumption, and the two most favorable tariffs (2.0A and 2.0DHA), an expense model is obtained for P0 and P1, as shown in Table 6. The first model, P0P1 2.0A, during billing 15 to 22 only has a mean deviation of ±0.32 € to the actual monthly billed behavior, validating its use as an estimate for subsequent billings. If we modify the model for the 2.0DHA tariff, we obtain the third column of Table 6 (P0 and P1 2.0DHA) that offers lower costs simulating a scenario in which residents prioritize comfort but would have contracted the 2.0DHA tariff. Next, we will compare both models for actual consumption to determine the savings generated by the HERMES system after its implementation.

From billing period 23 to 37, the average monthly savings in energy billed would be from 7.08 € to 11.58 €, i.e., a reduction of between 16.24% to 24.08% in energy billed compared to models without load shifting (see Table 6). If we consider taxes (excise tax of 5.11269632% for VAT of 21%: *1.0511269632*1.21), the average saving in each invoice would be from 9.00 € to 14.73 € (from 0.3 to 0.5 € per day) since the implementation of the HERMES system.

**Figure 21:**
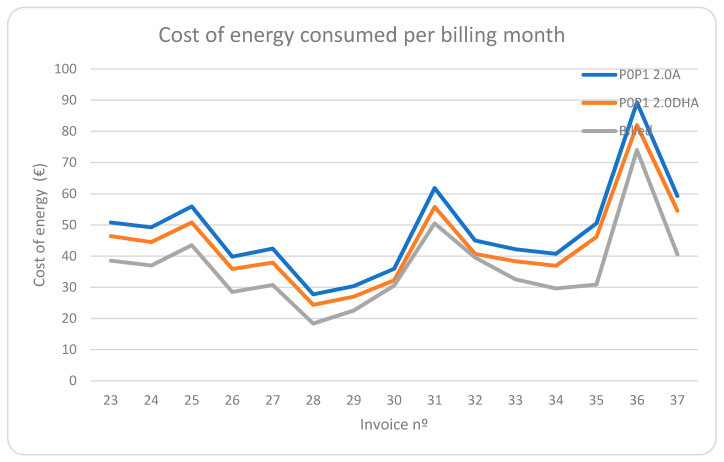
Cost of energy consumed (€) by billing months for the scenario without load shifting or habit adaptation (P0P1 series) and the actual cost billed since implementing the HERMES system.

**Figure 22:**
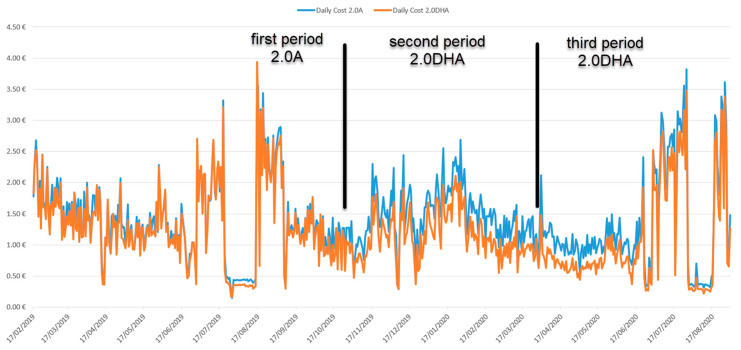
Comparison of the cost of energy consumed daily between the 2.0A (blue) and 2.0DHA (orange) tariffs from 17 February 2019 to 31 August 2020.

If we represent these data graphically, we obtain Figure 21:

Finally, in Figure 22, we compare the cost of energy consumed daily for the two regulated price tariffs, tariff 2.0A and 2.0DHA, from the first period to the third period.

We can see how graphically the cost in both tariffs is very similar during the first period. From the second period onwards, the 2.0DHA tariff was contracted, which implied a change in certain habits of the residents to adapt to the new tariff. In addition, from this second period onwards, the system already managed consumption and load shifting, which made it possible to optimize the time slots with lower prices, achieving a very significant reduction in the daily cost compared to the 2.0A tariff.

### 4.6. Billing Expenses in Absolute Values without Balancing

**Figure 23:**
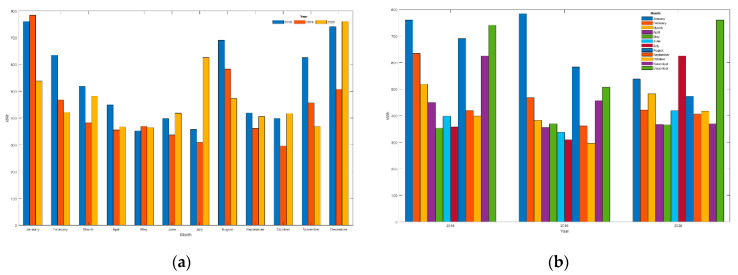
(**a**) For each month, the comparison of electricity consumed (kWh) is sorted by year. (**b**) For each year, a comparison of electrical energy consumed (kWh) is sorted by month.

**Figure 24:**
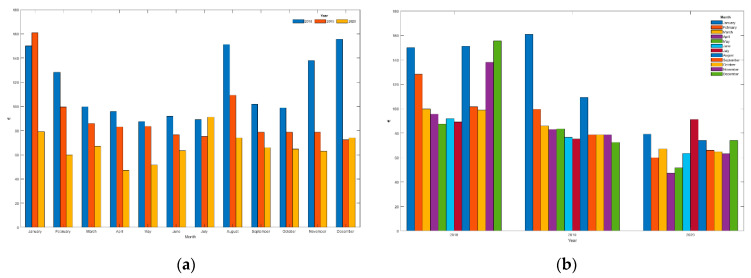
(**a**) For each month, a comparison of the amount of the electricity bill (€) is sorted by year. (**b**) For each year, a comparison of the amount of the electricity bill (€) is sorted by month.

Independently of the studies and models discussed above, we can conclude the savings analysis by detailing the bills issued by the electricity company, although in this case, the results are not balanced against price variations (tariff 2.0DHA: 2018: 0.1025 €/kWh; 2019: 0.0898 €/kWh; 2020: 0.0739 €/kWh), different annual temperature cycles (average Tmean: 2018: 18.2 °C; 2019: 18.8 °C; 2020: 19.2 °C) or different annual consumptions (total per year: 2018: 6346 kWh; 2019: 5211 kWh; 2020: 5644 kWh). However, it is of interest to show them given that the variations in conditions between 2019 and 2020 have not been very significant and yet show a remarkable reduction in bills even though the reduction in consumption has not been so significant (see Table 7 and Table 8, Figure 23 and Figure 24), mainly due to comfort requirements (higher consumption). Despite these demands, all months from the first period (31 March 2019 to 26 October 2019) present lower bills than the previous period (from 1 January 2018 to 31 March 2019), with a reduction of 18.3% where residents were unaware of their consumption details; as the first period progresses, the reduction in the bill is increasingly significant. This reduction is very striking with the entry of the second period (from 4 November 2019 to 28 March 2020). During this phase, the sum of the bills amounts to 357.1 € compared to 639.8 € during the same period a year earlier; the saving is 282.7 €, reducing 44.2% in the electricity bill. Finally, the bills from the third period (from 29 March 2020 to 31 August 2020) add up to 327.5 € compared to 427.7 € in the first period (reduction of 23.4%) or 515.3 € in the previous period (reduction of 36.5%) during those same months (from April to July), which shows the efficiency of the system able to continue optimizing periods when the system was partially implemented and already showing good performances as it was the first period. Table 7 (energy consumed) and Table 8 (monthly billing) also show the annual variations, including all periods.

The following figure shows a comparison of the data in Table 7:

The following figure shows a comparison of the data in Table 8:

Since the implementation of the system (a process developed during the first period), there has been practically no reduction in energy consumption in the home (see Figure 23), so comfort has not been sacrificed. However, the electricity bill has been reduced (see Figure 24); that is, the comfort of the residents has been maintained (and even improved) (thanks to the Wizard), and the loads have been shifted to reduce the monthly bill significantly.

The Assistant has significantly improved the residents’ sense of comfort by allowing them to voice-control most of the charges. Thanks to this positive impact, the additional (and primary) function of the Assistant of being able to transfer information to the residents (and to the system) to reduce the amount of the bills has been easily assimilated by the users, so the impact has been very positive and relevant, favoring the feeling of comfort and the reduction of the electric bill of up to 42%.

### 4.7. Consumption Estimation (Machine Learning)

**Figure 25:**
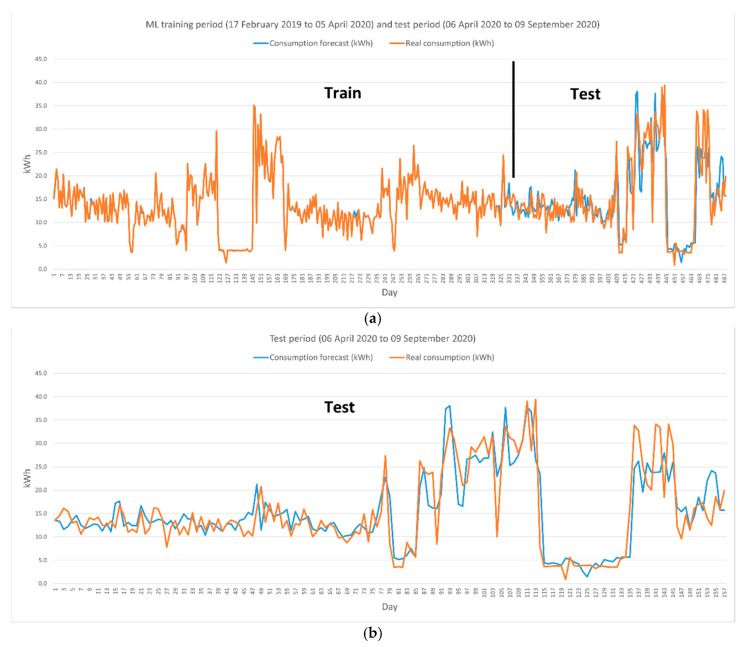
Comparison between estimated consumption and actual consumption in Wh. (**a**) ML Table (17 February 2019 to 5 April 2020) and test period (6 April 2020 to 9 September 2020). (**b**) Test period (6 April 2020 to 9 September 2020).

Finally, we show a comparison between what was consumed and the consumption estimate in Figure 25, which allows residents to detect habits that may increase spending when actual consumption consistently exceeds the estimate or beneficial habits when actual consumption is lower than the prediction.

For ML development, several regression algorithms were used to train the model. Given the characteristics of the data and the desired outcome, the algorithms offering the most accurate predictions were boosted decision tree regression (BDTR) and decision forest regression (FDR), as opposed to linear regression or neural network regression [93,94,95]. In our case, after multiple pieces of training with different data structures, the BDTR algorithm has provided excellent accuracy (coefficient of determination: 0.9842; relative absolute error: 0.1085; mean absolute error: 452.399) at the cost of moderate training times. The BDTR algorithm is very sensitive to overfitting, so care must be taken in setting up the algorithm.

The consumption forecast obtained by ML is very accurate because it handles a large number of variables, so if the same conditions are repeated, the consumption should be similar. Although there may be discrepancies, the long-term trend should show a high correlation between the forecast and the actual consumption, which made the BDTR algorithm an optimal candidate because it is based on the creation of a set of regression trees through boosting, which means that each tree depends on previous trees. The algorithm learns by adjusting the residual value of the trees preceding it, so boosting tends to improve accuracy by creating series of trees incrementally and selects the optimal tree by an arbitrary differentiable loss function.

The study data in this paper cover periods extending before, during, and after the confinement period due to COVID’19. Residents remained during the first confinement period (15 March 2020 to 20 June 2020) in the home and through mid-August 2020, with consumption increasing significantly during July due to high-temperature weather. In general, it was expected that consumers would be much higher than normal during the confinement period because the residents remain in the home all the time, which should translate into higher consumption. Figure 23 shows that the consumption from March to July 2020 is higher than the previous two years; however, the bills during that period (see Figure 24) were lower than the previous years (except July 2020). This highlights two relevant aspects, on the one hand, consumption should have increased significantly, but the system as a whole has been able to control these unfavorable conditions, and on the other hand, bills should have been much higher than in the same period of previous years, but again, the system has been able to manage the loads by reducing the energy impact to bills with lower amounts than in the previous two years. The system’s efficiency is very relevant, as, without it, we could have expected these bills to have increased very significantly.

## 5. Discussion

Intelligent energy management is a recurring and widely discussed topic in the scientific community. The continuous incorporation of new hardware and software elements is achieving increasingly complex and efficient goals. In this paper, we have presented a novel approach at the crossroads between energy management systems and Voice Assistants. The research is focused on residential environment but could be extended to energy communities, commercial buildings, or microgrids benefiting both customers (energy savings and comfort) and utilities (support of demand side management role in enhancing the flexibility of local energy systems). It combines energy management system, Voice Assistant, IoT, AI, and big data in a single ecosystem to create a novel Energy Management Expert Assistant that learns and adapts to users while improving system efficiency without sacrificing comfort. The system has been developed and implemented in a real pilot, allowing it to evaluate and optimize the decisions taken and improve during its implementation. This practical implementation has required a development that has been spread over two years. It integrates numerous IoT sensors and actuators, thus a large amount of data have been collected and stored in time series and relational databases. The implementation has been developed in three phases (P0–P1, P2, and P3) to optimize the development of the system. In the first period (P1), the habits of the residents were monitored, which made it possible to create a base model to optimize the decisions made by the system within acceptable comfort ranges for the users. The incorporation of the Virtual Assistant has maximized the results obtained. In this phase, we also optimized the best location and type of sensors and actuators to improve comfort and incentivize the participants. Two more phases were developed, being the third one where the system is already in full performance, and the best results are obtained. In this paper, we have presented the data up to this third period.

The work provides new developments in several lines of interest with real experimental results (not simulated) for which a measured deployment of sensors, actuators, as well as the development of IoT applications, recording of large amounts of data, visualization and processing of the data generated, modelling, ML, IoT intelligent environments, ES, and obtaining patterns has been required. It has been developed to obtain energy savings, cost reduction, comfort improvement, and social projection.

In addition to the above benefits, if this energy management system were widely adopted, it could provide interesting value-added elements for both users and utilities. Some of these elements could be: (1) adaptation of residents to routines suggested by the Wizard that allow to modify consumption habits and reduce the amount of bills, (2) load-shifting to the valley times, therefore (3) reducing consumption at peak times, (4) allowing the reduction of total peak demand for distribution grid congestion alleviation, (5) a more flexible response to demand from two levels of action: a first level that would be managed by our system (local) without significantly affecting the comfort of users, and a second level in which it is the aggregator or the utility (external system) which, through a demand response policy, act on the consumption of household appliances, potentially affecting the comfort of users, and (6) social work by reducing consumption and therefore emissions of greenhouse gases or assistance to specific groups with special needs served by the Assistant: elimination of barriers in the home, a sense of companionship, natural connection with the outdoors, information and advice on consumption, etc.

Finally, it should be noted that the ML data and its model have been published (see Data Availability Statement), and given the information it can generate, we believe it will be a fundamental tool for optimizing energy consumption and comfort as a future continuation of this work. Future research directions would focus on adding new elements of power generation, storage, demand response, power quality, greater flexibility of the system to shorten adaptation times for users and vice versa, and consumption prediction to optimize the use of these energy sources, minimize expenditure and maximize comfort. The Wizard will continue to be a fundamental element after the good results achieved during its use in the present work.

## Figures and Tables

**Table 1:** Classification of the main household appliances according to their load.

Uncontrollable Loads	Controllable Loads			
	Inelastic		Elastic	
	Uninterruptible Loads	Interruptible Loads	Variable Loads with Alteration of Comfort	Variable Loads without Altering Comfort
Television	Washing Machine	Electric Vehicle	Air Conditioning	Natural + artificial light
Sound equipment	Dishwasher	Phone Charger	Heating System	Automatic opening of windows
Computer	Dryer Machine	Battery/Energy Storage	Fan	
Fridge-freezer	Oven	Water Heater	Stove	
Lighting		Water Pump (Well, Pool)		
Standby		Vacuum Cleaner (robot)		
Microwave				
Vacuum Cleaner				
Iron				
Cooker pot				
Cooker Hood				
Hair dryer				
Toaster, Blender, Kettle				

**Table 2:** Programmable parameters associated with loads of each appliance. System managed control: “●” or “■”. Resident-managed control: “○” or “□”.

Appliance	Parameters	Description
Variable loads without altering comfort		
Automatic opening of roller shutters—Automatic opening of roller shutters	●○ % of shutter opening. ○ Activate or deactivate the automatic shutter opening control.	●○ Normally residents will control the % opening of the blinds, but if automatic opening control is active, the system will open the blinds based on outside natural light and whether or not residents are present in the room.
Variable loads with alteration of comfort		
■□ Climate control-Air Conditioning + Heating System	■□ On and off. □ Initial temperature □ Time in minutes until the system automatically applies a second regulation. □ Adjustment of the degree variation (+1, −1, +2, −2) for the second regulation. ■ Order of execution of the second regulation. □ Annulment of the Order of execution of the second regulation. ■ Automatic shutdown in the absence of residents for more than a specified time.	Residents set the temperature. The system resets the temperature (▲▼) after a few minutes to reduce consumption without affecting comfort. ■ Automatic shutdown in case there is no resident in the house (thanks to the GPS tracking controlled by the System) or the presence detector in the room does not detect movement for more than 1 h.
●○ Fan-Fan	●○ On and off. ●○ Power.	●○ Usually, users will control its on, off, and power, but the system can turn it off or lower its power if the contracted consumption limit in the electricity tariff is exceeded.
■□ Stove-Stove	■□ On and off. □ Power	■□ Normally users will control its on, off, and power, but the system can turn it off if the contracted consumption limit in the electricity tariff is exceeded.
Interruptible loads		
●○ Electric boiler	●○ On and off. ●○ 6 + 1 operating time slots. ○ Temperature targets for each activation band. ●○ Adjusting the water heating curve. ● Observation of permanent consumption. ● Long-term disconnection	●○ Absolute management of thermos operation by both residents and the system. ● Switched off during prolonged periods of absence of residents (vacations). Switched back on several days before return.
■□ Vacuum cleaner (robot)-Vacuum Cleaner (robot)	□ Switching on and off ■ Recharge control	■□ Normally users will control its power on and off, but a power-on time and recharge time can be programmed.
●○ Electric Vehicle ●○ Battery-Energy Storage ●○ Water Pump (Well, Pool)	●○ Recharge time (on/off).	●○ Recharging would take place at the cheapest hours. Note: Not applied or scheduled to the study dwelling in the article.
Uninterruptible loads		
■□ Washing Machine	■□ Power-on time. ■□ Permanent consumption observation.	■□ Manual or system-programmed ignition at the cheapest time between 7:00 and 11:00 AM.
●○ Dishwasher-Dishwasher.	●○ Power-on time. ●○ Permanent consumption observation.	●○ Manual or system-programmed ignition at the cheapest time for the next 12 h (24 h).
■□ Dryer Machine	■□ Power-on time.	■□ Manual or system-programmed ignition. Note: Not applied or programmed to the study dwelling in the article.
■□ Oven	■□ Power-on time.	●○ Manual or system-programmed ignition Note: Not applied or programmed to the article study dwelling.
Uncontrollable loads		
●○ Television ●○ Sound equipment ●○ Computer. ●○ Refrigerator/Fridge-freezer. ●○ Light Spots/lighting. ●○ Microwave ●○ Vacuum Cleaner ●○ Iron ●○ Cooker pot ●○ Cooker Hood ●○ Hair dryer ●○ Toaster ●○ Kettle ●○ Blender	○ On and off Observation of general consumption. Notification by the Assistant. ● Rate information to residents on an hourly, strip, and daily basis.	○ Manual switching on and off by residents. ● Warning of excessive consumption (via Assistant and Telegram) in the absence of residents or exceeding the contracted power limit. ● Notification (via Wizard, Telegram, and control panel) of the electricity tariff.

**Table 3:** IoT sensors and actuators in the main loads of the home.

Appliance	Sensors/Actuators	Description
Variable loads without altering comfort		
Automatic opening of roller shutters-Automatic opening of roller shutters	WiFi shutter switch	Allows raising and lowering of blinds with percentage function by programming
Variable loads with alteration of comfort		
Air Conditioning-Air Conditioning + Heating System	Universal Remote Control with WiFi and IR. Programmable. Temperature sensor in the room Presence sensor in the room System consumption sensor	Thanks to the controller, the System controls all the functions of the Air Conditioning and Heating System. The temperature, presence, and consumption sensor allows the system to perform a secondary adjustment.
Fan-Fan	IoT built-in from the factory	It is linked to the Assistant and the system for voice control and automation.
Stove-Stove	WiFi Smart Plug	On/off control.
Interruptible loads		
Electric water heater	WiFi Smart Plug with consumption and power measurement. Wifi temperature sensor inside the water tank.	The system controls the on, off, and actual temperature of the water in the tank.
Vacuum Cleaner (robot)-Vacuum Cleaner (robot)	Factory-integrated IoT. WiFi Smart Plug.	The system (or residents) can activate and deactivate it. The optimal recharging time is programmed via the smart plug.
Uninterruptible loads		
Washing Machine	WiFi Smart Plug with consumption and power measurement.	After being manually programmed, the System (or the residents) decides the switch-on time.
Dishwasher-Dishwasher	WiFi Smart Plug with consumption and power measurement. Door opening sensor.	After being manually programmed, the System (or the residents) decides the switch-on time.
Uncontrollable loads		
Television Sound equipment Computer Refrigerator/Fridge-freezer Light Spots/lighting Microwave Vacuum Cleaner Iron Cooker pot Cooker Hood Hair dryer Toaster Kettle Blender	-	Manual turn-on and turn-off by residents. -Warning of excessive consumption (via Assistant and Telegram) in case of absence of residents or exceeding the contracted power limit. -Notification (via Wizard, Telegram, and control panel) of the electricity tariff.

**Table 4:** Main voice interactions with the Assistant.

Command	Type (Residents Request Information/System Informs about Triggering Events)	Description
“turn on/off/regulate device”	Residents/System	Residents control more than 80 functions (turn on, turn off, raise the temperature by one degree) of the different devices connected in the home. In some cases, the system detects that a device has been switched on so that under certain conditions, it acts automatically to reduce consumption while maintaining comfort (e.g., it raises the cooling temperature by one degree after a few minutes of operation).
“price”, “power”, “consumption”, “daily consumption”, “cheapest washing machine/hour”...	Residents	Residents can ask at any time for data related to consumption and expenditure: Price or active power being consumed at that moment to know the impact of connected appliances, the next cheapest hours, the accumulated consumption per hour, daily or monthly.
“room temperature”, “outside temperature”, “thermos temperature”, “probability of rain”....		Residents can know the data from the sensors connected in the house through the voice assistant or the probability of rain to make decisions based on these conditions and the electricity tariff to reduce consumption and maintain comfort.
“Departure or arrival home” (GPS + ping Wifi + door sensor).	System	The system detects if a Resident arrives or leaves the house by issuing a welcome message or checking if there are devices or unwanted presences.
“Power warnings”	System	The System monitors the active power level, informing Residents if the contracted power limit is reached or exceeds 105%, which would incur penalties.
“Price and consumption/expense notices”	System	The System reports at each start of a time slot with a different energy price, except during night hours (peak, flat or off-peak). In the event of higher or lower than expected consumption, the reports and responses to automatic warnings and queries made by the Residents to the Assistant are modified.
“Notices on ways to save”	Residents/System	A compendium of tips with saving techniques. The advice offered is random unless an inappropriate use of an appliance is detected (e.g., forced turning on of the electric boiler or continued use of the washing machine during peak rate hours). The system has a calendar, so some responses and warnings change depending on whether it is a national holiday or a weekend, or if adverse weather conditions are expected, or a very high consumption prediction estimated by ML.
“Text-to-speech and social networks”	Residents/System	Residents can send any command to the Assistant through their mobile application by voice commands or by text through social networks that the Assistant receives and executes. The System uses social networks to send text and text-to-speech messages to Residents with the help of the Assistant.

**Table 5:** Phases of incorporation of HERMES system functionalities.

Periods		Validity (Day-Month-Year)	Incorporation of HERMES System Functionalities
P0	Previous	<31-03-2019	None
P1	First	31-03-2019 to 26-10-2019	Consumption information wizard
P2	Second	04-11-2019 to 28-03-2020	Consumption management and load shifting (electric boiler and washing machine). Change of optimal electricity tariff for the HERMES system.
P3	Third	29-03-2020 to 06-02-2021	Load shifting (dishwasher) and cooling temperature control

**Table 6:** Cost of energy consumed (€) by billing months for the scenario without load shifting or behavioral adaptation (P0P1 series) and actual billed cost. The data are divided into modeled and real data.

Billing Periods (Day-Month-Year)	Modeling		Real	
	P0 and P1 2.0A	P0 and P1 2.0DHA	Invoiced ^1^ (Energy)	Contracted Rate
Invoice 15: 09-03-2019 to 07-04-2019	44.22	40.52	44.26	2.0A
Invoice 16: 07-04-2019 to 07-05-2019	41.18	37.70	40.38	2.0A
Invoice 17: 08-05-2019 to 07-06-2019	41.08	37.57	40.78	2.0A
Invoice 18: 08-06-2019 to 06-07-2019	37.03	33.85	36.91	2.0A
Invoice 19: 07-07-2019 to 05-08-2019	35.71	32.75	35.05	2.0A
Invoice 20: 06-08-2019 to 06-09-2019	60.18	54.78	60.19	2.0A
Invoice 21: 07-09-2019 to 06-10-2019	37.91	34.53	37.67	2.0A
Invoice 22: 07-10-2019 to 03-11-2019	31.15	28.44	30.78	2.0A
Subtotal before Hermes (€)	**328.46**	**300.14**	**326.02**	
HERMES system implementation				
Invoice 23: 04-11-2019 to 09-12-2019	50.77	46.41	38.54	2.0DHA
Invoice 24: 10-12-2019 to 09-01-2020	49.25	44.50	36.99	2.0DHA
Invoice 25: 10-01-2020 to 07-02-2020	55.93	50.82	43.51	2.0DHA
Invoice 26: 08-02-2020 to 07-03-2020	39.82	35.86	28.50	2.0DHA
Invoice 27: 08-03-2020 to 10-04-2020	42.40	37.92	30.76	2.0DHA
Invoice 28: 11-04-2020 to 09-05-2020	27.74	24.36	18.38	2.0DHA
Invoice 29: 10-05-2020 to 06-06-2020	30.37	26.99	22.54	2.0DHA
Invoice 30: 07-06-2020 to 06-07-2020	35.95	32.25	30.56	2.0DHA
Invoice 31: 07-07-2020 to 08-08-2020	61.84	55.78	50.49	2.0DHA
Invoice 32: 09-08-2020 to 06-09-2020	45.00	40.73	39.57	2.0DHA
Invoice 33: 07-09-2020 to 06-10-2020	42.19	38.33	32.53	2.0DHA
Invoice 34: 07-10-2020 to 08-11-2020	40.75	36.91	29.62	2.0DHA
Invoice 35: 09-11-2020 to 07-12-2020	50.53	46.15	30.89	2.0DHA
Invoice 36: 08-12-2020 to 11-01-2021	89.31	82.04	74.02	2.0DHA
Invoice 37: 12-01-2021 to 06-02-2021	59.28	54.56	40.56	2.0DHA
Data from 04-11-2019 to 06-02-2021:				
Total (€)	721.13	653.61	547.46	
Average energy billed per month (€)	48.08	43.57	36.50	
Average monthly savings (%)	24.08%	16.24%		
Average monthly savings (€)	11.58	7.08		
Monthly savings with taxes (€)	14.73	9.00		
Average daily savings (€)	0.3859	0.2359		
Daily with taxes (€)	0.4909	0.3000		

^1^ Actual data provided by the electric company.

**Table 7:** Monthly energy billed: previous period P0; first period P1 (yellow); second period P2 (green); third period P3 (blue).

Month	Billed Monthly Energy (kWh)		
	2018	2019	2020
January	760	784	538
February	635	468	422
March	518	383	482
April	449	356	367
May	353	369	365
June	398	337	419
July	358	309	625
August	690	583	473
September	420	362	406
October	399	296	418
November	625	457	369
December	741	507	760
Total per year (kWh)	6346	5211	5644
Variation compared to 2018	0	−1135	−702
Variation compared to 2018 (%)	0%	−17.88%	−11.06%
Annual invoice (€)	1387.16	1082.32	801.19
Variation € compared to 2018 (%)	0%	−21.98%	−42.24%

**Table 8:** Monthly bill: previous period P0; first period P1 (yellow); second period P2 (green); third period P3 (blue).

Month	Monthly Amount (€)		
	2018	2019	2020
January	150.02	160.92	79.09
February	128.21	99.51	59.99
March	99.83	85.92	66.96
April	95.66	83.04	47.13
May	87.46	83.55	51.59
June	91.94	76.57	63.43
July	89.00	75.24	91.23
August	151.23	109.25	74.08
September	**101.68**	78.57	65.93
October	98.72	78.70	64.70
November	137.92	78.58	63.04
December	155.49	72.47	74.02 ^1^
Total per year (€)	1387.16	1082.32	801.19
Variation compared to 2018	0	−304.84	−585.97
Variation compared to 2018 (%)	0%	−21.98%	−42.24%
Annual energy billed (kWh)	6346	5211	5644
Variation kWh/year compared to 2018 (%)	0%	−17.88%	−11.06%

^1^ HERMES System upgrades from 20 December 2020 to 11 January 2021 for system maintenance (change from Raspberry Pi3B+ to 4B, upgrade to Raspbian Buster, Java 11, OpenHAB 3, fixed and removed security bugs, update of certain parts of the programming due to version changes and new syntax...).

## Data Availability

The Machine Learning and associated data presented in this study are available in Experiments from Azure AI Gallery (https://gallery.azure.ai/experiments accessed on 23 July 2021) under the title “Predicting the energy consumption of a house (BDTR-E1d)”. Public link: https://gallery.cortanaintelligence.com/Experiment/Predicting-the-energy-consumption-of-a-house-BDTR-E1d accessed on 23 July 2021.
